# Metabolic Patterns in *Spirodela polyrhiza* Revealed by ^15^N Stable Isotope Labeling of Amino Acids in Photoautotrophic, Heterotrophic, and Mixotrophic Growth Conditions

**DOI:** 10.3389/fchem.2018.00191

**Published:** 2018-05-31

**Authors:** Erin M. Evans, Dana M. Freund, Veronica M. Sondervan, Jerry D. Cohen, Adrian D. Hegeman

**Affiliations:** ^1^Department of Horticultural Science, University of Minnesota, Twin Cities, Saint Paul, MN, United States; ^2^Plant and Microbial Genomics Institute, University of Minnesota, Twin Cities, Saint Paul, MN, United States; ^3^Department of Plant and Microbial Biology, University of Minnesota, Twin Cities, Saint Paul, MN, United States

**Keywords:** stable isotope, nitrogen, *Spirodela polyrhiza*, duckweed, autotrophic, heterotrophic, mixotrophic, amino acids

## Abstract

In this study we describe a [^15^N] stable isotopic labeling study of amino acids in *Spirodela polyrhiza* (common duckweed) grown under three different light and carbon input conditions which represent unique potential metabolic modes. Plants were grown with a light cycle, either with supplemental sucrose (mixotrophic) or without supplemental sucrose (photoautotrophic) and in the dark with supplemental sucrose (heterotrophic). Labeling patterns, pool sizes (both metabolically active and inactive), and kinetics/turnover rates were estimated for 17 of the proteinogenic amino acids. Estimation of these parameters followed several overall trends. First, most amino acids showed plateaus in labeling patterns of <100% [^15^N]-labeling, indicating the possibility of a large proportion of amino acids residing in metabolically inactive metabolite pools. Second, total pool sizes appear largest in the dark (heterotrophic) condition, whereas active pool sizes appeared to be largest in the light with sucrose (mixotrophic) growth condition. In contrast turnover measurements based on pool size were highest overall in the light with sucrose experiment, with the exception of leucine/isoleucine, lysine, and arginine, which all showed higher turnover in the dark. K-means clustering analysis also revealed more rapid turnover in the light treatments with many amino acids clustering in lower-turnover groups. Emerging insights from other research were also supported, such as the prevalence of alternate pathways for serine metabolism in non-photosynthetic cells. These data provide extensive novel information on amino acid pool size and kinetics in *S. polyrhiza* and can serve as groundwork for future metabolic studies.

## Introduction

**Figure 1:**
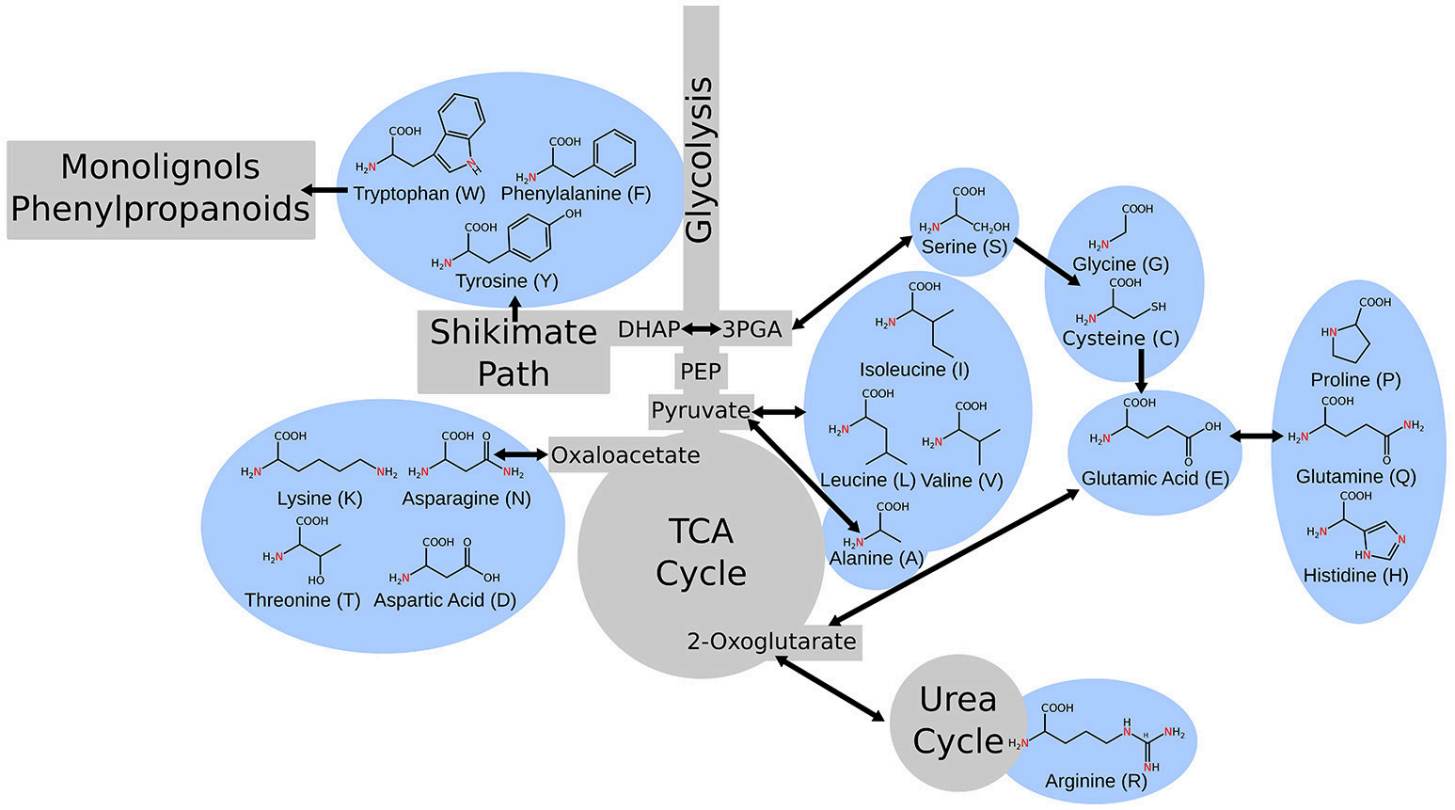
A simple schematic of central metabolism in the context of carbon flow showing the points where the metabolism of various amino acids intersect.

Primary metabolism in plants is responsible for providing the cell with fixed carbon, energy, reduced cofactors for cellular reactions, and all the building blocks for secondary metabolism and production of biomass. This portion of metabolism, including glycolysis, the citric acid cycle, the pentose phosphate pathway, amino acid metabolism, and fatty acid synthesis are relatively conserved across species (Peregrín-Alvarez et al., 2009). Amino acid metabolism is a unique portion of metabolism as it bridges carbon and nitrogen metabolism and amino acids are important intermediates in many metabolic processes. Alanine interacts with glycolysis and the citric acid cycle via pyruvate (Schulze-Siebert et al., 1984). Asparagine and its derivatives link through oxaloacetate (Lea and Fowden, 1975). Serine connects through photorespiration and feeds into one-carbon/folate metabolism (Ros et al., 2014). Arginine plays a key role in nitrogen storage and in the urea cycle (Witte, 2011). The aromatic amino acids feed into portions of secondary metabolism, namely phenylpropanoid metabolism, and lignin biosynthesis and tryptophan, specifically, provides a substrate for auxin biosynthesis (Maeda and Dudareva, 2012; Figure 1).

One of the most fascinating qualities of central metabolism in the context of plants is that they have the ability to run primary metabolism as both autotrophs and heterotrophs. The most basic examples of this are the differences between shoot (metabolic source) and root (metabolic sink) tissue (Ho, 1988; Sonnewald and Fernie, 2018) and the shifts in metabolism that occur with diurnal cycles when photosynthesis cannot occur (Geiger and Servaites, 1994). These changes have the potential to reveal how plants manage their resources such as fixed carbon from CO_2_ in the air, and nitrogen, minerals, and water from the soil. Understanding these plant resource allocation practices can have a potentially large impact on food and plant production (Sonnewald and Fernie, 2018) as the end products of central metabolism are the main components of biomass: starch, lipids/fats, and proteins are also the main nutrients people seek in food. The ability to manipulate these core metabolites/nutrients may carry more weight than before as consumer demands and federal regulations of food products are beginning to shift toward healthier macronutrient profiles including improved lipid profiles (Unnevehr and Jagmanaite, 2008).

Light and dark effects on amino acid pools have been well established for a number of years due to many studies carried out in the 1960s through the 1980s (Singh, 1998). These studies have assessed amino acid pool sizes in the context of whole plants over the day-night cycle. Each of these studies has shown strong variation in amino acid pool sizes between day and night. The general trend, however, showed that free amino acid pool sizes tended to be lower during the dark night hours (Noguchi and Tamaki, 1962; Bauer et al., 1977). This contrasts with studies of chlorophyll synthesis mutants in barley and corn which exhibit accumulation of almost all of the free amino acids (Maclachlan and Zalik, 1963; Shortess and Amby, 1979). Additional studies in *Zostera marina* root tissue indicated key amino acids, such as glutamate/glutamine and alanine having lower pool sizes in the dark (Pregnall et al., 1987). More recent studies on the transcriptional regulation of key enzymes involved in amino acid biosynthesis support these earlier light/dark amino acid studies (reviewed in Coruzzi, 2003).

Metabolic models are curated maps representing how the chemical reactions of metabolism are connected. Resent advances in using annotated genomes to build these networks in a number of plant species including *Arabidopsis thaliana* (Poolman et al., 2009; de Oliveira Dal'Molin et al., 2010a; Arnold and Nikoloski, 2014), corn (de Oliveira Dal'Molin et al., 2010b), and developing *Brassica napus* embryos (Schwender et al., 2003) have ignited an expansion in the field of metabolic flux and network analysis which seeks to estimate the quantitative flow of matter through metabolic reactions. Many of these advances have been in the realm of stoichiometric methods, such as flux balance analysis and they have been possible because of the emerging high-quality metabolic models (Sweetlove and Ratcliffe, 2011). These methods have been used to elucidate costs associated with amino acid biosynthesis under different nitrogen and metabolic growth conditions (Arnold et al., 2015), resource allocation during seed filling (Schwender et al., 2006), and overall metabolic patterns in central metabolism (Szecowka et al., 2013). However, these studies have had the advantage of having been conducted with species that have well-developed genomes as well as genome-scale metabolic models. In contrast, there are many organisms that do not have these well-developed system models.

The species of interest for this study is the common duckweed (*Spirodela polyrhiza*), a small aquatic angiosperm and a member of the family *Lemnaceae* which contains five genera: *Landolita, Lemna, Spirodela, Wolffia*, and *Wolffiella*. The *Lemnaceae* are monocotyledonous plants that reproduce primarily through asexual budding (Appendroth et al., 2013). They are also among the fastest growing angiosperms and are capable of doubling biomass in 16–24 h depending on the conditions (Peng et al., 2007). Additionally, they are able to accumulate high starch content of up to 75% of the dry weight (Reid and Bieleski, 1970; Xu et al., 2012b) and high-protein content. This ability to accumulate a large percentage of biomass as starch has led to some commercial interest in duckweed species as biofuel feedstock and it has been shown that it is possible to increase this starch content under nutrient stresses (Reid and Bieleski, 1970; Cui and Cheng, 2015) and also under certain light regimens (Yin et al., 2015). Duckweed species also grow readily on wastewater. A particular example of growth of three isolates on swine lagoon water (Bergmann et al., 2000; Cheng et al., 2002; Xu et al., 2011) highlights the possibility of mixing duckweed production into existing agricultural pipelines. Additional commercial interests for duckweeds include bioremediation (Oron, 1994) and recombinant protein production (Xu et al., 2012a). Because of the utility of duckweed for many uses it is going to be increasingly important to have an understanding of the full extent of metabolism, including metabolic rates/fluxes, static pool sizes, and information on how these respond to different growth and metabolic conditions such as differing light treatments.

In this study, we present a metabolic [^15^N]-labeling study of *S. polyrhiza* grown under three metabolic growth conditions: growth in light cycle on medium with supplemental sucrose (mixotrophic condition), growth in light cycle on medium without supplemental sucrose (photoautotrophic condition), growth in dark on medium with supplemental sucrose (heterotrophic condition). This growth set-up allows us to accomplish several objectives. First, our aim was to profile nitrogen metabolism and flow through amino acids and [^15^N]-labeling patterns over time to determine differences in the metabolic patterns between growth conditions. The second, aim was to estimate pool size and turnover information for amino acids in each condition and identify changes between growth conditions. The third aim was to determine if broad patterns in nitrogen flow exist between growth treatments that can be captured through clustering analysis. *S. polyrhiza* and other duckweeds have a history of use in stable isotopic labeling studies (Rhodes et al., 1981; Baldi et al., 1991; Rapparini et al., 1999) and can readily incorporate label from liquid growth medium. The [^15^N]-labeling study of *S. polyrhiza* facilitated the ability to measure amino acid pool sizes, turnover numbers, and adjusted pool sizes and to compare between experimental conditions. Here we lay the groundwork for further in-depth metabolic studies as well as reveal insights into central metabolism in duckweed.

## Materials and methods

### *S. polyrhiza* material and long-term culture maintenance

All duckweed cultures used in this work were clonally propagated *Spirodela polyrhiza* [ID number 7498 from the Rutgers Duckweed Stock Cooperative (http://www.ruduckweed.org/)] maintained on Schenk and Hildebrandt (SH) medium prepared from SH Basal Salt Mixture (Sigma-Aldrich St. Louis, MO) with 10% w/v sucrose and 1% agar with the pH adjusted to 5.8 using potassium hydroxide. Cultures were kept at 15°C, with a 15 h light cycle under white light from an LED array with light intensity of 17 μmol/m^2^•s.

### Experimental growth conditions

For experiments conducted in the light, actively growing duckweed cultures were maintained on sterile medium prepared from SH Basal Salt Mixture (Sigma-Aldrich St. Louis, MO) with the pH adjusted to 5.8 using potassium hydroxide, either with or without 10% w/v sucrose depending on experimental conditions. Cultures were maintained in a controlled growth environment for 4–6 weeks, under a 16/8 day/night cycle of cool white florescent light at an intensity of 67 μmol/m^2^•s at 22°C. Light was measured approximately at the level of the cultures. Cultures were determined to be saturated when the growth was covering the entire surface with little to no medium visible when viewed from above. Upon saturation the culture was transferred to labeled medium as described below.

For experiments conducted in the dark, actively growing duckweed cultures were maintained on sterile SH medium prepared from SH Basal Salt Mixture at pH 5.8 (Sigma-Aldrich St. Louis, MO) supplemented with 3.01 × 10^−3^ mM kinetin as used for *Lemna gibba* (Slovin and Tobin, 1982) in a dark box at room temperature under 2 min of red light every 8 h (10 μmol/m^2^•s) at room temperature. Cultures were only removed from the dark box under green light. When the cultures reached saturated growth, the culture was transferred to labeled medium for sampling as described below.

### [^15^N]-labeling experiment medium preparation

[^15^N]-labeled modified SH with a) no added sucrose; b) 10% w/v sucrose; c) 10% w/v sucrose and 3.01 × 10^−3^ mM kinetin, was prepared according to the specifications in Table S1. Nitrogen was provided as 80.7% K[^15^N]O_3_ and 19.3% [^15^N]H_4_ [^15^N]O_3_. The pH of the solution was adjusted to 5.8 with potassium hydroxide and autoclaved. The necessary iron components were added by syringe filter sterilization after the medium cooled to minimize precipitation of medium components.

### Duckweed transfer and sampling procedure

#### Experiments with a light cycle

Duckweed from actively growing cultures ± sucrose were decanted from the culture under aseptic conditions and washed with sterile distilled deionized water. Duckweed fronds were then sorted into five groups and transferred into either five 2 L Pyrex bottles with 300 mL of medium, or five 500 mL Pyrex bottles with 100 mL of medium. Once duckweeds were transferred, initial samples were taken from the extra plant material not needed for the labeling and transferred to pre-weighed 1.5 mL microcentrifuge tubes and snap frozen in liquid nitrogen. Samples were taken at the following time points: 0, 1, 2, 4, 8, 16, 32, 64, and 128 h. Additional samples were also taken at 15 and 30 min timepoints from cultures grown without added sucrose. Each sample consisted of at least three duckweed fronds. Snap frozen samples were stored at −80°C until extraction and LC-MS analysis. Two experiments were carried out for the light without sucrose conditions, which were started at different times in the day. One of these, started later in the day, was used for pool size calculation, and the other, started near the beginning of the light cycle in line with the other light experiment, was used to estimate label enrichment and estimate the kinetic model. This growth contained four replicates rather than five.

#### Experiments in the dark

The transfer and sampling procedures took place the same as for light cycle experiments with the following exceptions. Sampling was carried out in the dark under green light. Samples were taken at the following time points: 0, 15, and 30 min, and 1, 2, 4, 8, 16, 32, 64, and 128 h.

### Microscopy

Duckweed plants were grown in either light or dark conditions for at least 2 weeks. When cultures reached saturation individual duckweed fronds were removed and thin layers of leaf tissue were scraped off with a clean razor and put onto a microscope slide. Brightfield and fluorescence images were taken at 1,000x magnification with Leitz Laborlux D (Stuttgart, Germany) fluorescence microscope. Fluorescence images were acquired with an N2.1 filter to visualize chlorophyll [excitation wavelength range: 515–560 nm (green); emission wavelength: >590 nm (red)]. Image J was used to make montages, overlays, and to add scale bars (Schindelin et al., 2012, 2015).

### Sample extraction

Frozen sample weights were taken. Samples were kept on dry ice and up to 1.5 mL/mg sample fresh weight cold 70% isopropanol was added to each sample. Samples were then homogenized in a Geno/grinder tissue homogenizer at 1,500 rpm for 5 min. Sample homogenate was then centrifuged at room temperature for at least 3 min and supernatant was decanted into a fresh microcentrifuge tube and stored at −80°C until mass spectral analysis.

### Dry residue analysis

100 or 200 μL of sample extract was added to a pre-weighed 1.5 mL microcentrifuge tube and the samples were then dried under vacuum and the weight of the residue was determined. Samples were reconstituted to a concentration of 2 mg sample extract/mL in 70% isopropanol and stored at −80°C until mass spectral analysis.

### LC-MS analysis

#### LC sample preparation

Extracted samples were removed from storage and centrifuged at room temperature for at least 2 min to settle particulate matter. Samples were then loaded into LC-MS autosampler vials. Fresh plant extracts were diluted 1:10 in 70% isopropanol. Dried extract samples were diluted to a concentration of 1 μg/μL in 70% isopropanol in the light experiment without supplemental sucrose and the dark experiment and to a concentration of 0.9 μg/μL in the light with sucrose experiment. A commercially available [^15^N]/[^13^C]-labeled cell free mixed amino acid standard (Sigma-Aldrich St. Louis, MO) containing all proteinogenic amino acids was added to dry residue samples for a final concentration of 0.02 mM. All measurements were carried out on five biological replicates except in the light with sucrose experiment, which had only three replicates.

#### LC-MS analysis

For each sample, 1 μL was injected onto a SeQuant ZIC-cHILIC column, 3 μm particle size, 100 × 2.1 mm using an Ultimate 3000 UHPLC system coupled to a Q-Exactive quadrupole-Orbitrap hybrid mass spectrometer (Thermo Fisher Scientific, Waltham, MA) with a heated electrospray ionization source. A 20 min gradient at a flow rate of 0.4 mL/min with mobile phase A (0.1% formic acid in water) and B (0.1% formic acid in acetonitrile) with the following gradient: −2–0 min: 90% B, 0 min: 85% B, 18 min: 40% B, 18–20 min: 40% B. MS analysis used the following settings: full scan mode in positive ionization with a scan range of 50–750 *m/z*, a resolution of 70,000, a target automatic gain control of 1 × 10^6^, and a maximum fill time of 200 ms. Data were collected using Thermo *Xcalibur* software version 4.0. Amino acid identity was verified through comparison of retention times and accurate mass to a commercially available mixed amino acid standard. The amino acid isomers of leucine and isoleucine are quantified together because of the inability to separate these with the chromatography system employed.

### Data analysis

#### Exact mass calculation and retention time determination

Exact masses for both labeled and unlabeled amino acids were calculated using the University of Wisconsin—Madison Biological Magnetic Resonance Data Bank exact mass calculator (http://www.bmrb.wisc.edu/metabolomics/mol_mass.php). Retention times and exact mass measurements of all amino acids were confirmed using amino acid standard H (Sigma-Aldrich, St. Louis, MO).

#### MS file conversion and data extraction

Data files were converted from *.RAW* files to *.mzXML* files using the “msconvert” function of *Proteowizard* (Kessner et al., 2008) prior to input into *R*. Data from each amino acid was extracted through use of a pair of scripts developed in the Hegeman lab currently available on GitHub (https://github.com/orgs/HegemanLab files “metabolite turnover” and “clustering”). Briefly, we utilized the *ProteinTurnover* (Fan et al., 2016) and the *XCMS R* packages (Smith et al., 2006; Tautenhahn et al., 2008; Benton et al., 2010) to extract amino acid extracted ion chromatograms (EICs) for each amino acid isotopomer. These data were then used to generate labeling patterns tracking the decay of the unlabeled isotopomer (M0).

#### Data modeling

Labeling patterns for M0 output by the clustering script were then used to model the labeling pattern of each amino acid via the “nls” function in *R*. The broom package was used to clean the data for export. The model implemented was a variation of the model by Yuan et al. (2006) for modeling a first-order decay of the unlabeled (M0) isotopomer:

$$\begin{array}{l} M_{0}=\left(1-c\right)e^{-kt}+c \end{array}$$

Where *t* is the labeling time in hours, k is the turnover constant, and c is a constant that defines the plateau of the labeling curve. Models were generated using time points 0–16 h except for the following cases that used 0–32 h: lysine in the dark grown experiment and 0–64 h: tryptophan in the light grown experiment without supplemental sucrose and alanine, arginine, threonine, and tryptophan in the dark grown experiment. Histidine was omitted from all modeling and valine was omitted from the light without sucrose and dark with sucrose experiments due to low data quality. All models were estimated using five biological replicates except in the light without sucrose experiment, which had only three replicates.

#### Clustering

*K*-means clustering was performed using *R*, time points between 0 and 32 h with *k* selected to maximize the between-group variation and to minimize the within group variation. The Hartigan-Wong algorithm was used to form clusters based on Euclidean distance. Further clustering was carried out based on the pool sizes adjusted for turnover. The number of clusters used for these analyses were the same as used for the first analysis except in the case of the light with sucrose experiment where four clusters rather than 3 were used.

#### Estimation of pool size, pool size corrected for turnover, and active pool size

Pool sizes in all experiments were estimated from the initial time point to provide insight into the amount of available amino acid in each condition. Pool sizes were estimated relative to the labeled internal standard added to dry residue samples. All numbers for pool size carry the units μmol/mg sample extract residue.

The *k*-values from each model was used to estimate a flux value, denoted as pool size adjusted for turnover, for each amino acid as follows:

$$\begin{array}{l} f_{AminoAcid}=k_{AminoAcid}\times P_{AminoAcid} \end{array}$$

Where *f*_*AminoAcid*_ is the flux estimate through an amino acid, *k*_*AminoAcid*_ is the turnover number modeled for an amino acid, and *P*_*AminoAcid*_ is the initial pool size estimated for an amino acid. All flux dimension numbers or turnover corrected pool sizes carry units of μmol/mg sample extract residue/hour.

Pool sizes were corrected to approximate an “active pool” as observed in illuminated Arabidopsis rosettes (Szecowka et al., 2013) and maize (Arrivault et al., 2016). The c component in the models were taken as an approximation of the proportion of the pool not active in metabolism and the pool size estimates were corrected as follows:

$$\begin{array}{l} P_{Active}=P_{Initial}-\left(P_{Initial}-c\right) \end{array}$$

Where *P*_*Active*_ is the estimated corrected pool size, *P*_*Initial*_ is the originally estimated pool size, and *c* is the modeled plateau for each amino acid.

#### Pairwise statistical comparisons

Estimates for pool size and turnover adjusted pool size were compared across the three pairs of experimental conditions: light with sucrose vs. light without sucrose, light with sucrose vs. dark with sucrose, and light without sucrose and dark with sucrose. Comparisons were made via two-tailed student's *t*-test assuming unequal variance except where a *F*-test indicated equal sample variances in which case an equal variance test was applied.

## Results

### [^15^N]-labeling of *S. polyrhiza* amino acids in phototrophic, heterotrophic, and mixotrophic growth conditions

**Figure 2:**
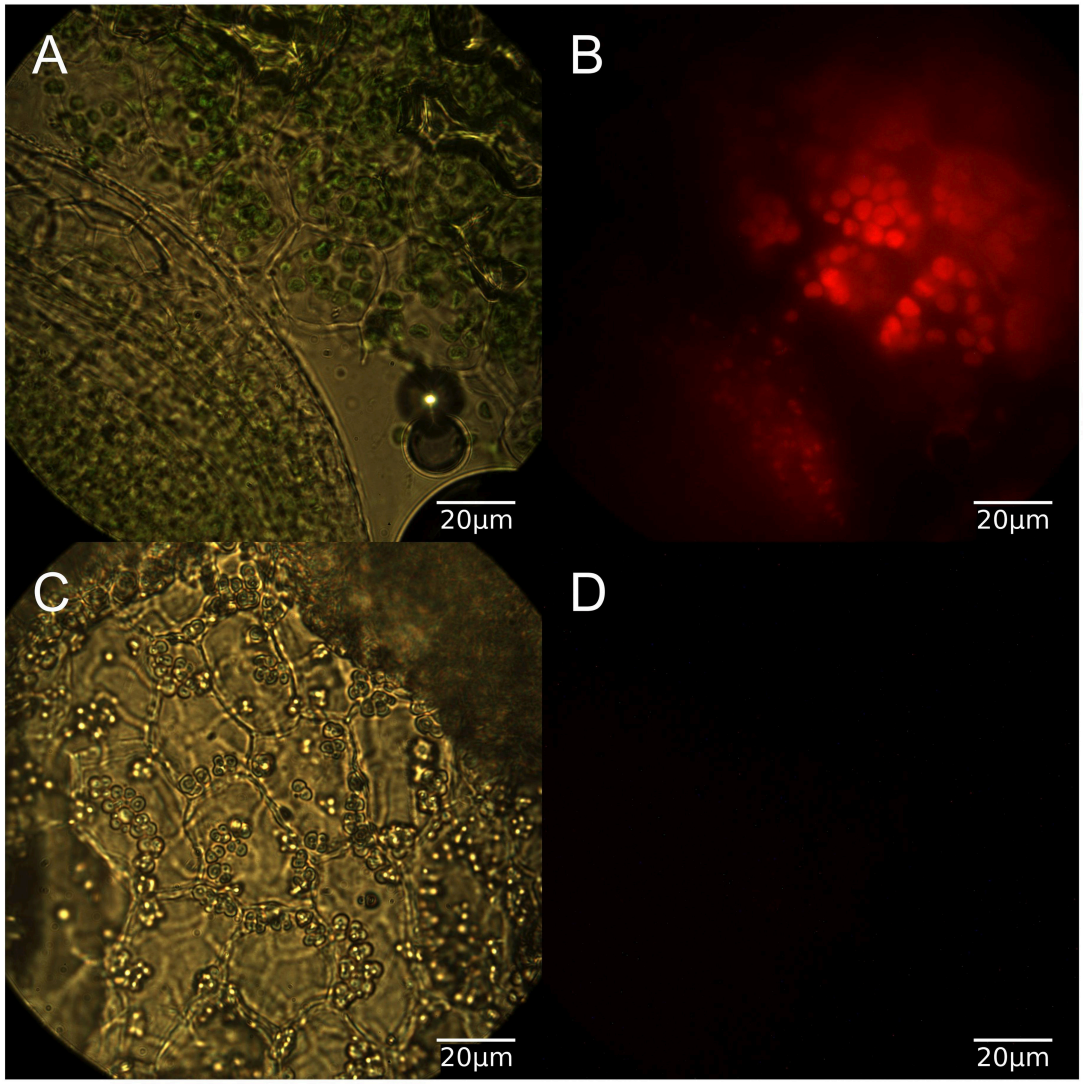
**(A)** Brightfield microscopy of light grown duckweed at 100x, **(B)** Red fluorescence image of light grown duckweed, **(C)** Brightfield image of dark grown duckweed, **(D)** Red fluorescence image of dark grown duckweed.

In order to confirm the physiological effects of the light conditions duckweed grown for at least 2 weeks in light or dark conditions were sectioned and imaged under both brightfield and red fluorescence (Figure 2). Light grown samples exhibited normal chlorophyll autofluorescence and as expected dark grown samples had no detectable chlorophyll autofluorescence. [^15^N]-labeling was conducted once the growth conditions were confirmed as phototrophic, heterotrophic, and mixotrophic based on chlorophyll content. Fifteen amino acids were identified by utilizing LC-MS and retention time windows; their isotopomer molecular ion masses are in Table S2.

### [^15^N]-label incorporation models for individual amino acids

**Figure 3:**
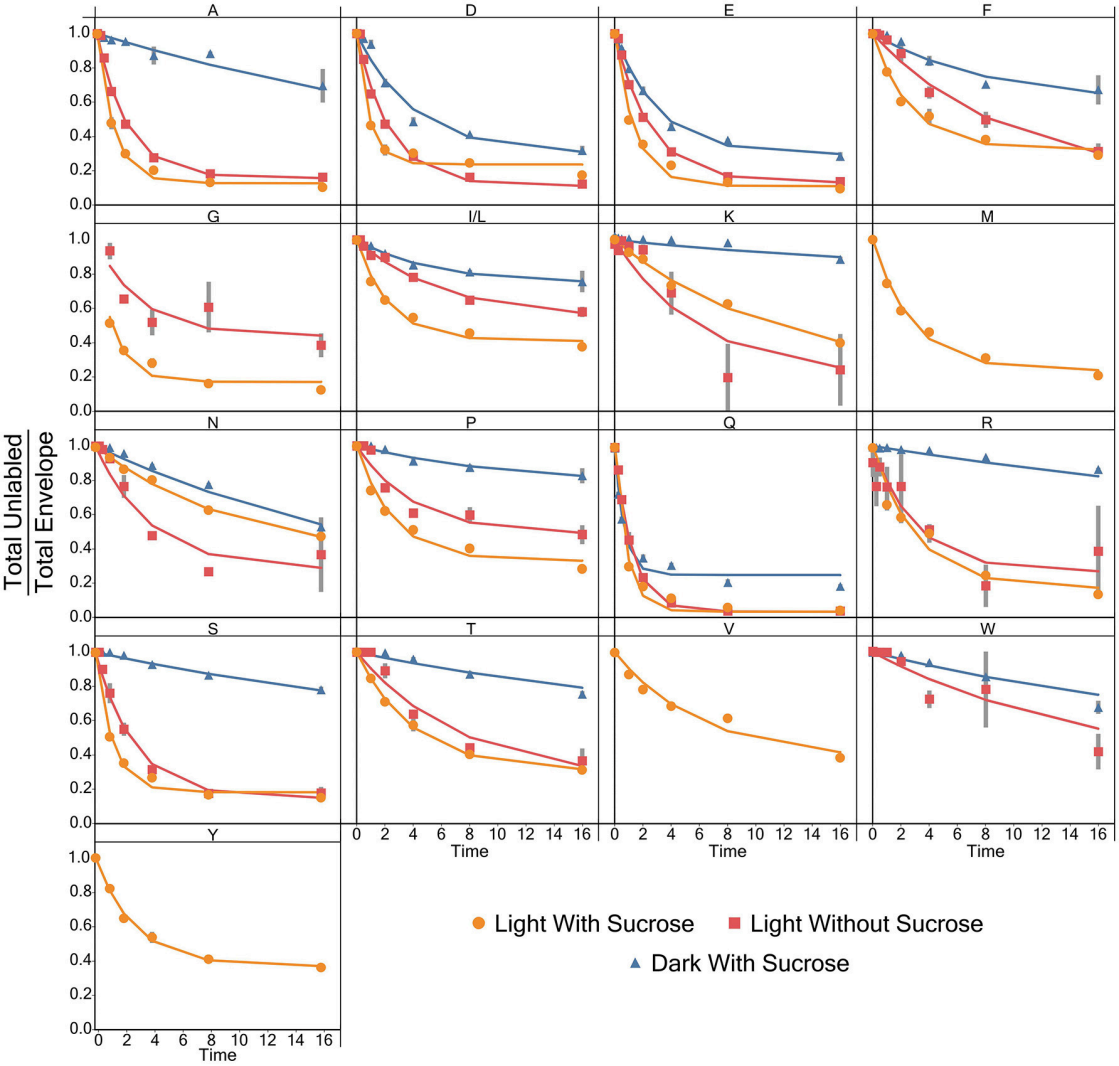
A graph showing the average ratio of the unlabeled isotopomer to the total isotopic envelope for each amino acid in each experiment. The lines represent models generated in R. Error bars represent ± 1 standard error.

Models for the incorporation rate of [^15^N]-label into each individual amino acid were generated. Figure 3 contains a visual representation of each model and the values for the parameters of the models are listed in Table 1. General labeling patterns can be seen for all amino acids as well as between each experimental condition. Most amino acids exhibit a plateau or leveling-off phase around the 16 h time point with some amino acids, most notably glutamate, taking longer to reach that plateau. Dark grown samples generally exhibited slower turnover and higher plateau values with several amino acids, including alanine, asparagine, serine, and threonine not appearing to reach fully discernable plateau values within the timeframe measured Overall, the trends observed by modeling the rate of [^15^N]-incorporation indicate there are both dramatic and subtle trends that are seen when duckweed is grown in phototrophic, heterotrophic, and mixotrophic conditions.

**Table 1:** The modeled values for turnover number (*k*) and the [^15^N] labeling plateau value *c* for amino acids measured in *S. polyrhiza*.

**Amino acid**	**Experiment**	***k*-value**	**Standard error (k)**	***c*-value**	**Standard error (c)**
Alanine (A)	Light with sucrose	0.85	0.05	0.13	0.01
	Light without sucrose	0.47	0.03	0.16	0.02
	Dark with sucrose	0.03	0.01	0.15	0.09
Arginine (R)	Light with sucrose	1.16	0.11	0.24	0.01
	Light without sucrose	0.43	0.03	0.11	0.02
	Dark with sucrose	0.25	0.03	0.30	0.03
Asparagine (N)	Light with sucrose	0.70	0.05	0.11	0.02
	Light without sucrose	0.39	0.02	0.13	0.01
	Dark with sucrose	0.32	0.02	0.29	0.01
Aspartic acid (D)	Light with sucrose	0.38	0.03	0.32	0.02
	Light without sucrose	0.11	0.02	(0.15)	0.10
	Dark with sucrose	0.12	0.04	0.59	0.08
Glutamic acid (E)	Light with sucrose	0.78	0.05	0.17	0.01
	Light without sucrose	0.32	0.10	0.44	0.06
	Dark with sucrose	–	–	–	–
Glutamine (Q)	Light with sucrose	0.44	0.027	0.41	0.01
	Light without sucrose	0.16	0.02	0.54	0.03
	Dark with sucrose	0.19	0.05	0.74	0.03
Glycine (G)	Light with sucrose	0.09	0.02	0.22	0.08
	Light without sucrose	0.17	0.09	(0.20)	0.18
	Dark with sucrose	0.04	0.02	0.80	0.06
Histidine (H)	Light with sucrose	0.44	0.03	0.41	0.01
	Light without sucrose	–	–	–	–
	Dark with sucrose	–	–	–	–
Isoleucine (I)/Leucine (L)	Light with sucrose	0.35	0.02	0.24	0.01
	Light without sucrose	0.26	0.07	0.28	0.08
	Dark with sucrose	0.04	0.002	0.07	0.02
Lysine (K)	Light with sucrose	0.1	0.01	0.34	0.05
	Light without sucrose	0.25	0.05	0.48	0.04
	Dark with sucrose	0.09	0.05	0.77	0.07
Phenylalanine (F)	Light with sucrose	0.39	0.03	0.33	0.01
	Light without sucrose	0.82	0.05	0.03	0.02
	Dark with sucrose	1.51	0.11	0.25	0.01
Proline (P)	Light with sucrose	1.17	0.07	0.03	0.01
	Light without sucrose	0.32	0.15	0.27	0.12
	Dark with sucrose	0.02	0.004	0.40	0.07
Serine (S)	Light with sucrose	0.32	0.04	0.17	0.04
	Light without sucrose	0.36	0.03	0.15	0.03
	Dark with sucrose	(0.03)	0.02	(0.45)	0.24
Threonine (T)	Light with sucrose	0.84	0.04	0.18	0.01
	Light without sucrose	0.14	0.03	0.25	0.08
	Dark with sucrose	0.06	0.005	0.54	0.03
Tryptophan (W)	Light with sucrose	0.25	0.02	0.30	0.02
	Light without sucrose	0.06	0.03	0.30	0.13
	Dark with sucrose	0.04	0.01	0.46	0.04
Tyrosine (Y)	Light with sucrose	0.16	0.021	0.37	0.04
	Light without sucrose	–	–	–	–
	Dark with sucrose	–	–	–	–
Valine (V)	Light with sucrose	0.36	0.02	0.37	0.01
	Light without sucrose	–	–	–	–
	Dark with sucrose	–	–	–	–

*All values had a statistical goodness of fit of p < 0.01 except where otherwise noted in parentheses*.

### Estimation of amino acid pool size and pool size adjusted for turnover

**Figure 4:**
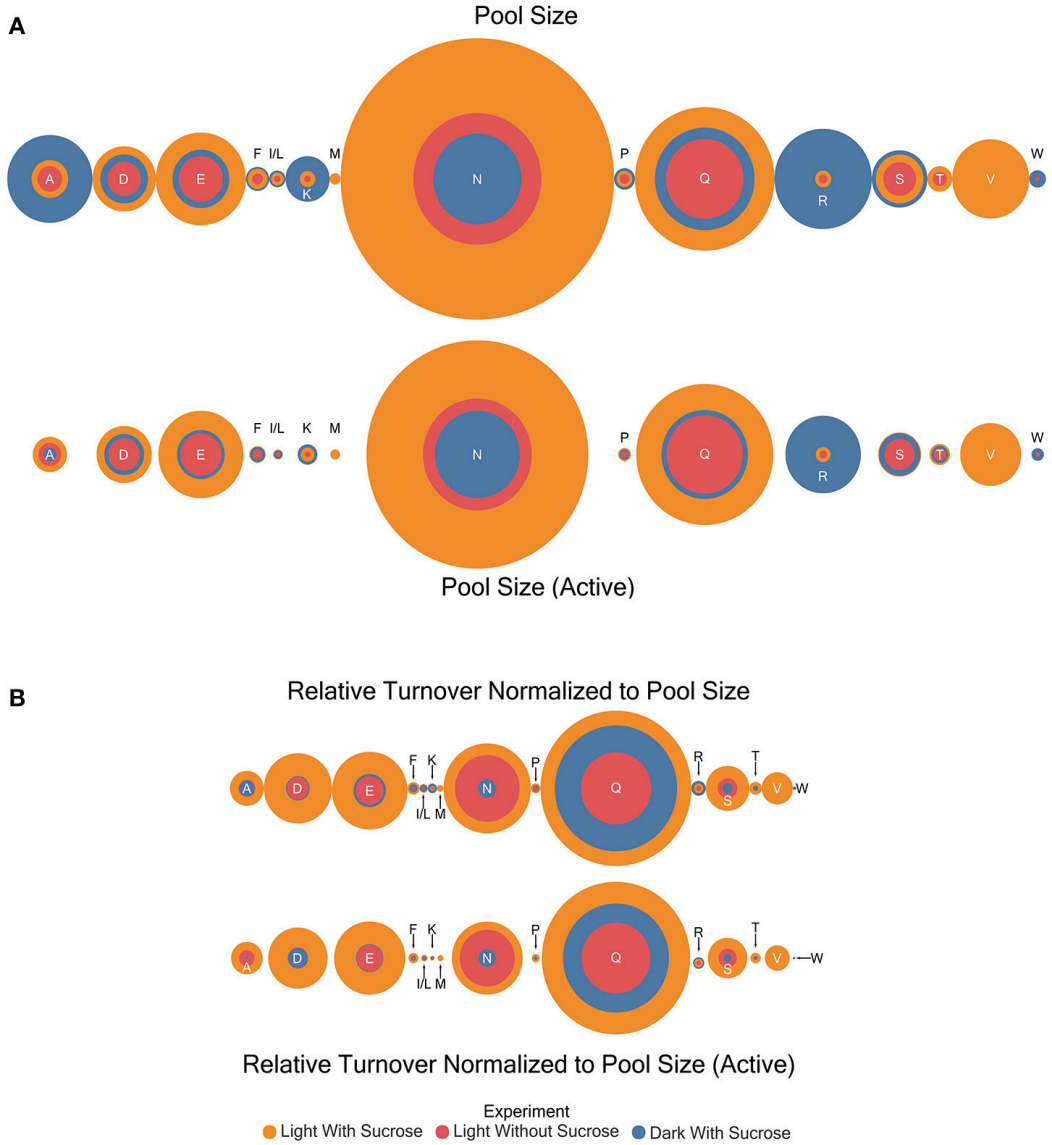
Circle area plots showing a visual representation of relative amino acid pool sizes (μmole) and turnover correct for pool size (μmole/hr) in each experiment. Colors indicate the experiment as shown. Circle areas are proportional to the magnitude of the estimate indicated. **(A)** Relative amino acid pool size and active pool size estimates **(B)** Results for turnover normalized to pool size for total and active portions.

Pool sizes in μmol/mg sample extract residue were estimated by comparison to an internal standard (Table S3). General patterns were observed through the experiments with the dark with sucrose condition exhibiting the largest pool sizes in almost every case where an amino acid was observed in the experiment (Figure 4A). However, the following amino acids are the exception: aspartic acid, glutamate, glycine, asparagine, and glutamine where the light with sucrose treatment exhibited larger pool sizes than other conditions. The light without sucrose treatment exhibited either the smallest or an intermediate pool size in every amino acid. Pool sizes can be explained to be either active or inactive. Active metabolite pools are those participating in metabolic reactions, while inactive pools are not and may be sequestered in a vacuole or otherwise unable to participate in metabolic reactions (Szecowka et al., 2013). Therefore, in addition to total pool sizes a total active pool size was estimated for each amino acid. For our purposes the active pool size was estimated by correcting the estimated pool size with the plateau value (*c*) of each model. Interestingly, following this correction the light with sucrose condition carried the largest pool size for all amino acids with the exception of lysine and arginine.

The turnover rates (estimated *k*-values) were multiplied by the initial pool size estimates for each amino acid to give a dimension of flux (μmol compound/unit time). This quantity is not a true flux value as it will encompass all enzymes and reactions involved in nitrogen transfer through each amino acid. The utility of the estimation is to allow direct comparison of turnover rates between amino acids that may have dramatically different pool sizes. Turnover rates adjusted for pool size in this way in contrast to regular pool size estimates were higher in the light with sucrose experiments in almost all cases (Table S3 and Figure 4B). The exceptions were isoleucine/leucine, lysine, and arginine which both showed larger turnover values in the dark. Similar to the pool size estimation the light without sucrose treatment exhibited either the smallest or an intermediate pool size in every case. However, the light with sucrose and light without sucrose measurements for asparagine were nearly identical.

Turnover rates were also corrected using the active pool size and the results are summarized in Table S3 and Figure 4B. These results largely mirror the uncorrected total pool sizes with a few interesting differences. Lysine showed a compressing of the differences between estimates with the dark grown pool now slightly larger than the light with sucrose pool. In several cases the differences observed in pool sizes between the dark grown and light were reduced to the point it became visually indistinguishable. Most notably in aspartic acid, glutamate, proline, and asparagine. In addition, there was a drastic reduction in the alanine turnover corrected for pool size in the dark grown experiment.

### Pairwise statistical comparison of pool size and turnover adjusted for pool size

Pairwise statistical comparisons were made between each sample pair (Table S4). Many experimental pairs, despite showing appreciable graphical difference did not show statistical significance at least to the *p* < 0.05 level. This may be due to large sample variances in many of the pool size measurements. The most significant differences in pool size were present in alanine between the two light experiments and the dark grown experiment as well as asparagine between the light with sucrose and the dark grown experiment. The results in alanine are preserved in the active pool estimates, while the results for asparagine are not. Additionally, differences in the pool size for proline between the two light experiments emerge when the pool sizes are corrected for a possible active pool. The turnover rates corrected for pool size show the most significant difference in phenylalanine and asparagine between the light without sucrose and the dark grown experiment and in serine between the two light experiments. When an active pool is considered, the differences in phenylalanine become less significant while the differences in asparagine and serine remain significant.

### Amino acids expressed labeling patterns that could be captured by clustering analysis. cluster composition was not conserved when *k*-means clustering was applied to turnover rates adjusted for pool sizes

**Figure 5:**
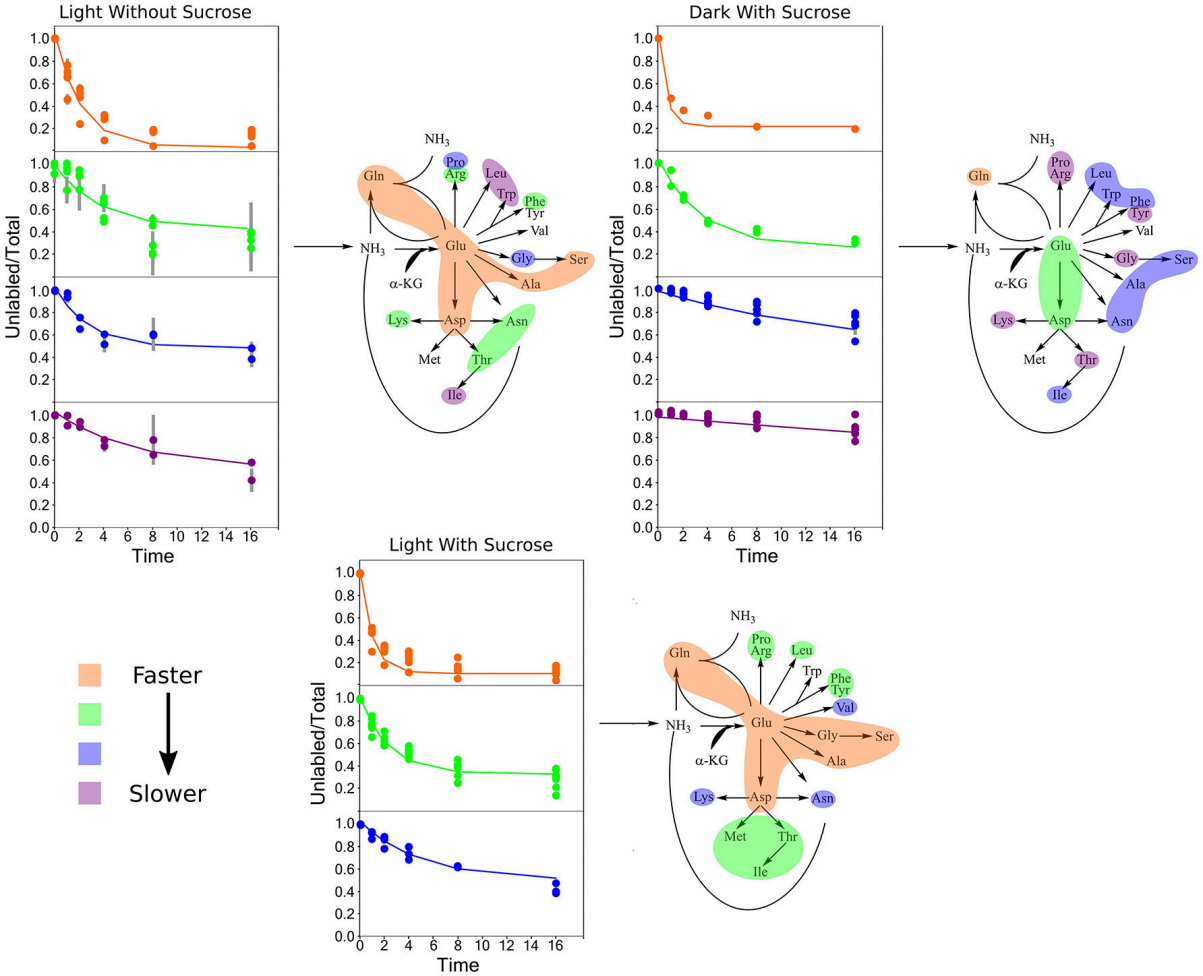
Average labeling patterns following the decay of the unlabeled isotopomer over time for each experiment. The lines are the models generated by *R* and the error bars represent ±1 SE. Maps adjacent to each set of graphs represent the flow of nitrogen among amino acids. The colored bubbles correspond to the clusters in the above figure highlighting similar isotopic exchange rates for the members of a cluster.

Clustering analysis was performed using a *k*-means clustering method to assess the similarities that may be present in the label incorporation for amino acids (Figure 5). The appropriate number of clusters (*k*_*clusters*_) was determined empirically by minimizing the within group sum-of-squares variation and maximizing the between group sum-of-squares variation. A *k*_*clusters*_ of 3 or 4 was found to be most appropriate depending on the experiment. Broad patterns were seen repeated between treatments using this method. There were distinct patterns in labeling patterns that could be seen between the growth treatments. Glutamine consistently showed the fastest turnover in all experiments, closely followed by glutamate and aspartate. Both light treatments had very similar rapidly turning over clusters consisting of glutamine, glutamate, aspartate, serine, and alanine. However, this grouping is broken up in the dark treatment where glutamine maintained a rapid turnover closely followed by glutamate and aspartate while a number of amino acids involved in carbon and nitrogen central metabolism, including asparagine, serine, and alanine displayed lower turnover and clustered separately from glutamate and aspartate. Each cluster was modeled by a non-linear regression (Table 2).

**Table 2:** A summary of the exponential decay models for each cluster.

**Experiment**	**Cluster**	***k*-value**	**p (k)**	**SE**	**c (constant)**	**SE**	**p (c)**
Light	1	1.002	^***^	0.05	0.179	0.01	^***^
With	2	0.437	^***^	0.02	0.380	0.01	^***^
Sucrose	3	0.205	^***^	0.03	0.530	0.04	^***^
	1	0.472	^***^	0.04	0.110	0.03	^***^
Light	2	0.128	^***^	0.05	0.000	0.25	–
Without	3	0.360	^***^	0.10	0.529	0.06	^***^
Sucrose	4	0.149	–	0.08	0.547	0.15	^***^
	1	1.659	^***^	0.12	0.278	0.02	^***^
Dark	2	0.286	^***^	0.03	0.302	0.03	^***^
With	3	0.061	^**^	0.03	0.514	0.19	^***^
Sucrose	4	0.008	–	0.08	0.000	9.26	–

Statistical significance is indicated by asterisks with the following levels represented:

*** *p < 0.005*,

** p < 0.01, and

− *p > 0.05*.

**Figure 6:**
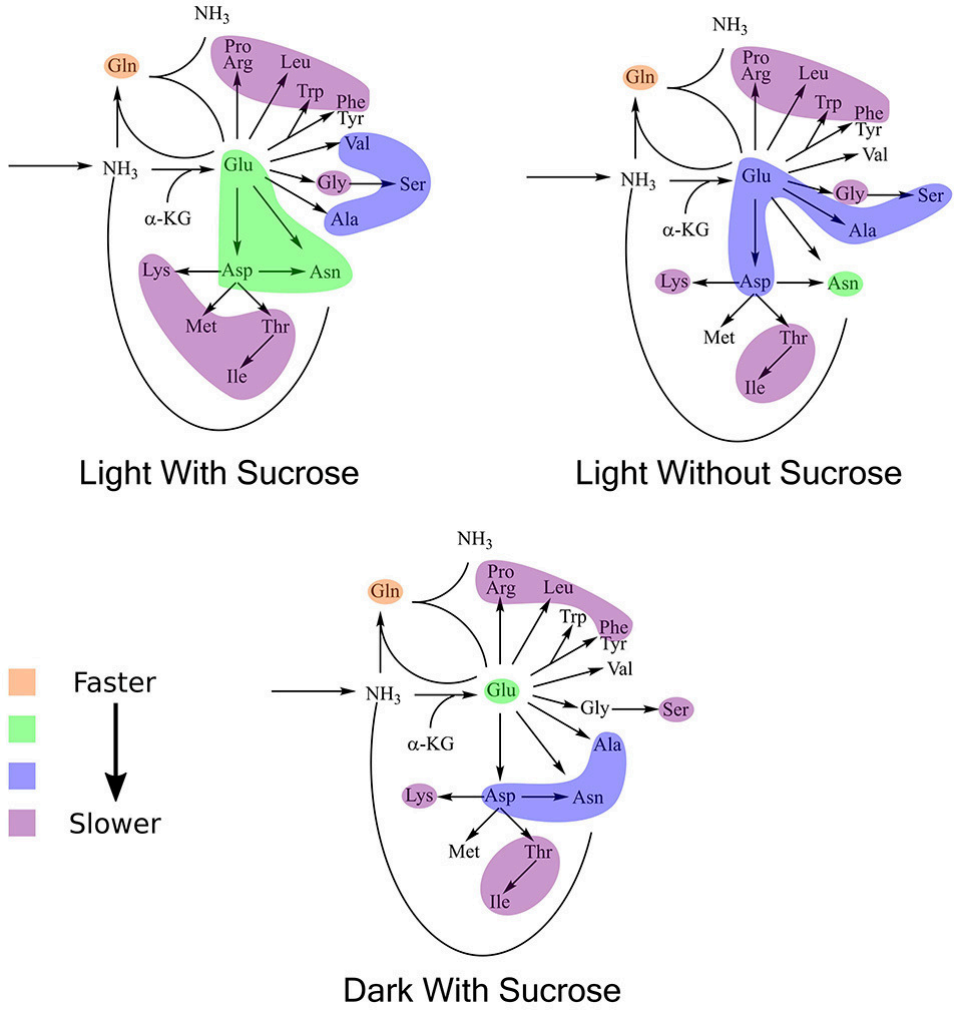
Results of *k*-means clustering for estimates of turnover normalized to active pool size. Colors of each bubble represent the same cluster positions (1–4) and relative exchange rates as in Figure 5.

*K*-means clustering was also performed using the estimated turnover rates adjusted for pool size for each amino acid individually. Exponential decay models for each amino acid and the clusters generated when considering turnover adjusted for pool size mapped onto a representation of amino acid nitrogen flow are shown in Table 2 and Figure 6, respectively. Several amino acids that seemed to be relatively “core” in the previous analysis (glutamine, aspartate, glutamate, and asparagine) retained their rapid turnover. Some amino acids that grouped into apparently rapid turnover segments in the first two analyses, notably glycine and arginine in the light experiments, were shown to have much lower numbers when pool sizes were taken into consideration. This demonstrates that modeling based on the pattern of labeling can be a useful way to categorize metabolites into broad metabolic groups without needing to individually model each amino acid in turn. However, this approach may convolute some details and may also be affected to a certain extent by inactive pools or plateaus in the labeling pattern and might also not reflect a full picture of the quantity of material moving through a given metabolite.

## Discussion

We used stable isotope labeling to model the kinetics of amino acids grown under three different light and/or carbon source conditions. Two conditions used a light cycle, either with or without 10% w/v sucrose in the growth media and one condition grown in the dark with 10% w/v sucrose in the growth medium. Growing an angiosperm plant under these three different conditions adds a considerable amount to the existing body of research involving stable isotope labeling *in planta* (Freund and Hegeman, 2017). Previous studies involving the labeling of whole plants generally have involved plants grown exclusively in heterotrophic conditions (Kikuchi et al., 2004; Szecowka et al., 2013). Photoautotrophic, mixotrophic, and heterotrophic growth conditions have all been investigated in the microalgae *Chlamydomonas reinhardtii* (Boyle and Morgan, 2009), however, there is a limited number of studies investigating whole plants grown and labeled under these three conditions. In particular we lay the groundwork for future intensive labeling studies for *S. polyrhiza* by demonstrating experimental and analytical frameworks that can be adapted to [^13^C]-labeling through the use of [^13^C]O_2_ or [^13^C]-labeled sugars.

The three growth conditions used in this study reflect fundamentally different metabolic modes, which in turn has an affect on the growth of the plant. This difference in plant status can be seen at both macroscopic and microscopic levels (Figure 2) as plants grown in light show normal coloration and chlorophyll autofluorescence while those grown in the dark show a bleached color and lack any sign of chlorophyll autofluorescence while still retaining plastid structures.

Labeling kinetics of each individual amino acid also shows interesting patterns. After analyzing and inspecting the data we chose to model the decay of the unlabeled isotopomer following Yuan et al. (2006) with an added term to account for the visible plateau reached by almost all of the amino acids by about 16 h. Each of these models, with few exceptions, has a very high goodness of fit level (*p* < 0.001) despite somewhat large standard errors in some cases. Though as noted in a similar analysis carried out by Szecowka et al. (2013) this may be due to an averaging effect across compartments, which could not be resolved in our current analysis. As described above models were generated using the time 0–16 h points for almost all amino acids. However, for certain amino acids, most notably many in the dark grown experiment the apparent plateau was not fully realized until closer to the 64 h time point, if at all.

**Figure 7:**
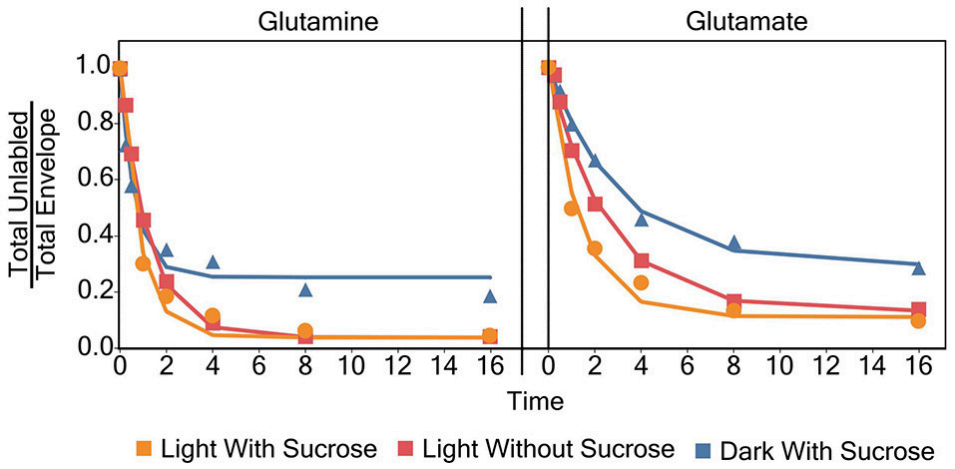
Individual models for glutamate and glutamine for each experiment.

This failure to reach a plateau can be seen in Figure 7 for glutamine. Both light experiments have models and labeling patterns that follow each other closely. However, the dark grown experiment begins to deviate from the model after the 16 h time point. This pattern continues for several amino acids in the dark grown condition, including: aspartate, glutamate, phenylalanine, isoleucine/leucine, lysine, proline, and serine. There are several possibilities that could account for this deviation from the initial models seen in the dark grown samples. Rather than a plateau/inactive pool, it would be simplest to model a two-phase labeling. This model would consist of an initial (fast) labeling, similar to our initial estimate followed by a slower second phase as (1) the larger-than-estimated active pool is completely washed out or (2) certain sub-pools/compartments with lower turnover begin to take up label. In addition, some irregularities can be observed in the labeling patterns, again in Figure 7, and as discussed later regarding metabolic clusters, where glutamine appears to label at a faster rate than glutamate, which seems novel as glutamate is the first entry point for nitrogen during uptake. It is uncertain what causes this labeling pattern, but it is possible that glutamine, which possesses multiple nitrogen atoms, may exhibit two different turnover coefficients, causing one nitrogen atom, most likely in the side chain, to label much more slowly than the alpha nitrogen.

We also used comparison of the time zero samples to a labeled standard to achieve estimates of pool size for most of the amino acids measured. No similar estimates have been carried out in any duckweed species to date to our knowledge making this a unique and useful data set. Several clear patterns emerged in these pool size estimates including the dark experiment showing larger pool sizes in alanine, phenylalanine, isoleucine/leucine, lysine, arginine, serine, threonine, and tryptophan. This result is notable, as many of these amino acids have been shown to accumulate in chlorophyll-deficient mutants during recovery of a normal pigment phenotype (Maclachlan and Zalik, 1963; Shortess and Amby, 1979). That we see the same patterns between dark grown and light grown with sucrose as was previously seen with some chlorophyll mutants indicates that the differences we are observing likely do come from a difference in autotrophic vs. heterotrophic status of the cell.

In addition to estimating total pool size, we also estimated an active pool size based on the observed plateaus in each labeling pattern. These active pools were defined as the total pool size minus a part of the pool size proportional to the modeled plateau. When this estimation was taken into account many of the amino acids displaying larger pool sizes in the dark no longer displayed larger pool sizes with respect to the light with sucrose treatment, with the exception of lysine, arginine, and serine. This result shows larger inactive pools, overall, in the dark grown experiments. This is significant particularly in asparagine, which is known to be a particularly prevalent nitrogen storage molecule (Lea et al., 2007), and one that is sensitive to the carbon status of the plant as well as arginine, which is generally a primary nitrogen storage molecule in the roots (Winter et al., 2015). As arginine pool sizes seem largest in the dark this could be an indication that metabolism in dark grown duckweed is functioning more like sink tissues such as roots.

Turnover rates were normalized to pool size by multiplying the rate by pool size estimates for each amino acid. By making this adjustment we are able to estimate a quantity with the dimensions of a flux parameter (moles/unit time) that can help better inform the results that we observed in the labeling patterns. While the turnover rates for many amino acids were noticeably slower in the dark, we can see that relatively larger pool sizes of these samples result in more moderate differences between treatments when considering the pool size normalized turnover rate values. This is consistent with the idea that those plants have a much more robust nutrient supply having carbon available through both photosynthetic carbon fixation and through supplemental sucrose in the medium. The notable exceptions to this are arginine and lysine which both show higher pool size adjusted turnover values in the dark. These pool size adjusted turnover rates were also calculated for the estimated active pools and yielded similar results. The one exception is the large decrease in pool size normalized turnover rate when estimated with the active pool size in alanine.

One of the most biologically interesting results is that of serine and the high level of statistical significance found for the difference between pool size adjusted turnover rate in the light experiments with and without sucrose and also between the light without sucrose and dark grown experiments. This is an even more notable result because of a recent interest in serine metabolism. The main source of serine biosynthesis in most conditions is the glycolate pathway (Douce et al., 2001; Ros et al., 2014). This pathway is associated with photorespiration and is thus not expected to be a contributor to serine turnover in conditions where photorespiration is low or nonexistent, such as in a root or an etiolated plant as in the dark grown condition potentially leading to lower turnover overall.

Before attempting individual modeling of amino acids we employed clustering analysis to identify broad patterns in amino acid labeling without the need to calculate individual models. This met with some success. However, when investigating a metabolic system, the most important measure is going to be the pathway flux. While we have not estimated a true flux through each amino acid for reasons outlined above pool size adjusted turnover rates give us a measurement with the dimensions of flux. When similar clustering analysis is carried out on these normalized rate values general patterns are still observed. However, many clusters now contain only one metabolite giving far less meaningful results. Even with this many of the patterns that are seen in the turnover adjusted for pool size, especially areas of relatively rapid turnover can still be seen. Though, there are some notable changes such as asparagine showing considerably higher turnover than was estimated in the light with sucrose condition through labeling pattern alone. Thus, while useful, due to the fact that they cannot fully capture fluxes and other more quantitative information a clustering-type analysis may be more useful when attempting to investigate metabolic dynamics where full identification and quantification of the involved metabolites is not possible, such as with portions of secondary or specialized metabolism.

## Conclusion

In this study stable isotope labeling was used to investigate labeling patterns, pool sizes, and turnover in *S. polyrhiza* under three growth conditions corresponding, roughly to full photoautotrophic growth, mixotrophic growth, and full heterotrophic growth and demonstrated growth of *S. polyrhiza* in the dark for the first time as well as providing the first amino acid pool size estimates for *S. polyrhiza*. These data established the clear labeling plateau and likely presence of metabolically inactive or less active pools in experiments with a light cycle. They also showed that correcting for active pool sizes could, in some cases, have a large effect on comparisons between experiments. We showed that pool sizes are not sufficient to make full metabolic comparisons and that adding a flux dimension, whether via full metabolic flux analysis or the use of turnover number corrected for pool size, can highlight differences that would be otherwise missed in simply considering pool size or turnover number alone.

Labeling patterns in each condition highlighted some interesting known (asparagine pool sizes in the dark) and emerging (serine turnover adjusted pool sizes) trends, particularly, the results regarding serine support the function of photorespiratory pathways as the most efficient/highest turnover mechanism of serine metabolism as we see pool accumulation in conjunction with low turnover in heterotrophic growth conditions. Labeling patterns were also captured by *k*-means clustering analysis. When pool sizes and turnover numbers were considered it became clear that many differences apparent when using those metrics could be masked when only considering the labeling pattern. However, the agreement that does exist between labeling pattern-derived clusters and turnover normalized for pool size indicate that application of clustering techniques based on labeling pattern could be useful for situations where many compounds are unknown, such as investigation of secondary metabolism.

## Data availability

The datasets generated and analyzed for this study can be found in University of Minnesota Digital Conservancy Data Repository: http://hdl.handle.net/11299/193599.

## Author contributions

EE wrote the manuscript and was primarily responsible for carrying out labeling experiments, sample collection, LC-MS analysis, and data analysis. DF assisted in design of labeling experiments and provided the LC-MS method. DF and VS assisted in carrying out labeling experiments and in LC-MS data collection. VS assisted in data analysis. JC and AH provided funding and project direction. All authors read, edited, and approved the manuscript.

### Conflict of interest statement

The authors declare that the research was conducted in the absence of any commercial or financial relationships that could be construed as a potential conflict of interest.
