# Supplementary material for: Metabolic Patterns in Spirodela polyrhiza Revealed by 15N Stable Isotope Labeling of Amino Acids in Photoautotrophic, Heterotrophic, and Mixotrophic Growth Conditions
Source: Front Chem. 2018 May 31;6:191. doi: 10.3389/fchem.2018.00191 (PMC5990592; doi:10.3389/fchem.2018.00191)
Supplement: Supplementary file 1 [file Table_1.DOCX]

Supplementary Material

Metabolic patterns in *Spirodela polyrhiza* revealed by ^15^N stable isotope labeling of amino acids in photoautotrophic, heterotrophic, and mixotrophic growth conditions

Erin Evans, Dana M. Freund, Veronica M. Sondervan, Jerry D. Cohen, Adrian D. Hegeman^*^

*** Correspondence:** Adrian D. Hegeman: hegem007@umn.edu

## Supplementary Tables

**Table S1.** Final concentration for all components of the [^15^N]-labeled growth medium.

| **Medium Component** | **Concentration (mM)** |
| --- | --- |
| K[^15^N]O_3_ | 21.88 |
| CaCl_2_ × 2H_2_O | 1.36 |
| MgSO_4_ × 7H_2_O | 1.62 |
| [^15^N]H_4_[^15^N]O_3_ | 2.61 |
| KH_2_PO_4_ | 2.61 |
| H_3_BO_3_ | 8.09•10^-02^ |
| MnSO_4_ | 6.62•10^-02^ |
| ZnSO_4_ | 6.19•10^-03^ |
| Na_2_MoO_4_ | 4.86•10^-04^ |
| CoCl_2_ × 6H_2_O | 4.20•10^-04^ |
| KI | 6.02•10^-03^ |
| CuSO_4_ × 5H_2_O | 8.01•10^-02^ |
| FeSO_4_ (or NaFe-EDTA) | 9.87•10^-02^ |
| Na_2_– EDTA (only With FeSO_4_) | 5.95•10^-02^ |
| Kinetin (dark experiments only) | 3.01•10^-3^ |

**
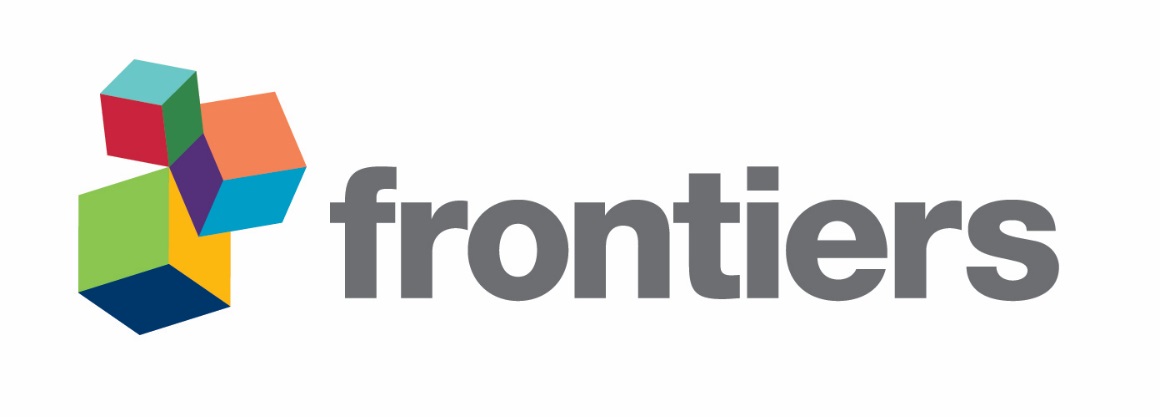
**
