# Supplementary material for: Metabolic Patterns in Spirodela polyrhiza Revealed by 15N Stable Isotope Labeling of Amino Acids in Photoautotrophic, Heterotrophic, and Mixotrophic Growth Conditions
Source: Front Chem. 2018 May 31;6:191. doi: 10.3389/fchem.2018.00191 (PMC5990592; doi:10.3389/fchem.2018.00191)
Supplement: Supplementary file 2 [file Table_2.DOCX]

Supplementary Material

Metabolic patterns in *Spirodela polyrhiza* revealed by ^15^N stable isotope labeling of amino acids in photoautotrophic, heterotrophic, and mixotrophic growth conditions

Erin Evans, Dana M. Freund, Veronica M. Sondervan, Jerry D. Cohen, Adrian D. Hegeman^*^

*** Correspondence:** Adrian D. Hegeman: hegem007@umn.edu

## Supplementary Tables

**Table S2**. Amino acid retention time ranges for each experiment and estimated exact mass values for each isotopomer

| **Amino Acid** | **Retention Ranges (Min)** | **M+H** | **M+1** | **M+2** | **M+3** | **M+4** | |
| --- | --- | --- | --- | --- | --- | --- | --- |
| Alanine (A) | Light With Sucrose: 4.55-5.8 Light Without Sucrose: 4.8-5.4 Dark With Sucrose: 4.8-5.4 | 90.0549 | 91.0520 | ------ | ------ | ------ |  |
| Arginine (R) | Light With Sucrose: 15.9-17.5 Light Without Sucrose: 14.8-16 Dark With Sucrose: 14-16 | 175.1189 | 176.1160 | 177.1130 | 178.1101 | 179.1071 |  |
| Asparagine (N) | Light With Sucrose: 7.6-8.6 Light Without Sucrose: 7.6-8.6 Dark With Sucrose: 7.6-8.6 | 133.0607 | 134.0578 | 135.0548 | ------ | ------ |  |
| Aspartic Acid (D) | Light With Sucrose: 7.4-8.9 Light Without Sucrose: 10.2-13 Dark With Sucrose: 12-16 | 134.0448 | 135.0418 | ------ | ------ | ------ |  |
| `Glutamic Acid (E)` | Light With Sucrose: 7.15-8.55 Light Without Sucrose: 7.2-8 Dark With Sucrose: 7.2-8 | 148.0604 | 149.0575 | ------ | ------ | ------ |  |
| Glutamine (Q) | Light With Sucrose: 6.9-8.3 Light Without Sucrose: 7-7.8 Dark With Sucrose: 7-7.8 | 147.0764 | 148.0735 | 149.0705 | ------ | ------ |  |
| Glycine (G) | Light With Sucrose: Light Without Sucrose: 6.1-6.7 Dark With Sucrose: 5.5-7 | 76.0393 | 77.0363 | ------ | ------ | ------ |  |
| Histidine (H) | Light With Sucrose: 6.1-7.4 Light Without Sucrose: 15.4-16.8 Dark With Sucrose: 15.4-16.8 | 156.0768 | 157.0738 | 158.0708 | 159.0679 | ------ |  |
| Isoleucine (I)/ Leucine (L) | Light With Sucrose: 2.6-3.8 Light Without Sucrose: 2.7-3.2 Dark With Sucrose: 2.7-3.2 | 132.1019 | 133.0989 | ------ | ------ | ------ |  |
| Lysine (K) | Light With Sucrose: 16.4-18 Light Without Sucrose: 15.4-16.6 Dark With Sucrose: 14.8-16.6 | 147.1128 | 148.1098 | 149.1069 | ------ | ------ |  |
| Phenylalanine (F) | Light With Sucrose: 2.65-3.85 Light Without Sucrose: 2.9-3.4 Dark With Sucrose: 2.9-3.4 | 166.0863 | 167.0833 | ------ | ------ | ------ |  |
| Proline (P) | Light With Sucrose: 3.75-5.05 Light Without Sucrose: 4-4.5 Dark With Sucrose: 4-4.5 | 116.0706 | 117.0676 | ------ | ------ | ------ |  |
| Serine (S) | Light With Sucrose: 7.95-9.5 Light Without Sucrose: 8.1-8.8 Dark With Sucrose: 8.1-8.8 | 106.0499 | 107.0469 | ------ | ------ | ------ |  |
| Threonine (T) | Light With Sucrose: 5.9-7.3 Light Without Sucrose: 6.1-6.8 Dark With Sucrose: 6.1-6.8 | 120.0655 | 121.0626 | ------ | ------ | ------ |  |
| Tryptophan (W) | Light With Sucrose: 2.95-4.15 Light Without Sucrose: 3.25-3.55 Dark With Sucrose: 3.25-3.55 | 205.0972 | 206.0942 | 207.0912 | ------ | ------ |  |
| Tyrosine (Y) | Light With Sucrose: 4.5-5.75 Light Without Sucrose: 4.8-5.3 Dark With Sucrose: 4.8-5.3 | 182.0812 | 183.0782 | ------ | ------ | ------ |  |
| Valine (V) | Light With Sucrose: 2.95-4.3 Light Without Sucrose: 1.8-2.1 Dark With Sucrose: NA | 118.0863 | 119.0833 | ------ | ------ | ------ | |

**
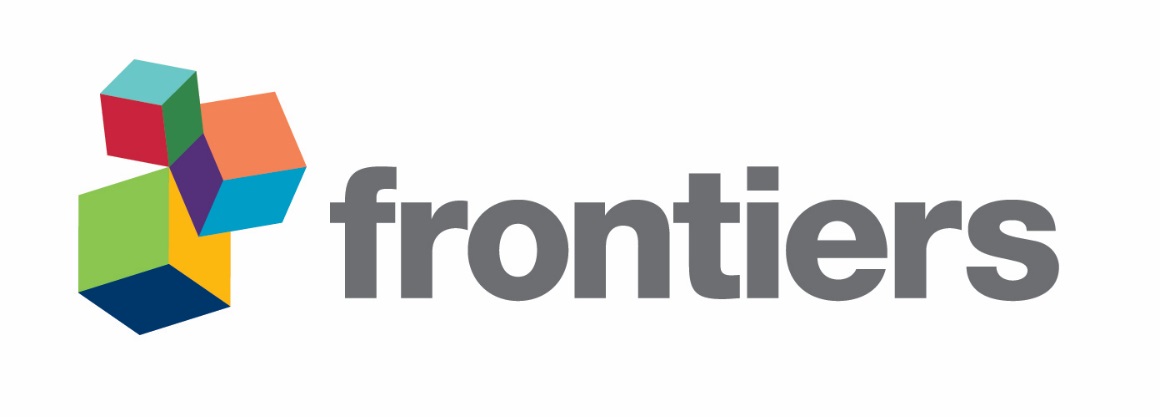
**
