# Supplementary material for: Metabolic Patterns in Spirodela polyrhiza Revealed by 15N Stable Isotope Labeling of Amino Acids in Photoautotrophic, Heterotrophic, and Mixotrophic Growth Conditions
Source: Front Chem. 2018 May 31;6:191. doi: 10.3389/fchem.2018.00191 (PMC5990592; doi:10.3389/fchem.2018.00191)
Supplement: Supplementary file 3 [file Table_3.DOCX]

Supplementary Material

Metabolic patterns in *Spirodela polyrhiza* revealed by ^15^N stable isotope labeling of amino acids in photoautotrophic, heterotrophic, and mixotrophic growth conditions

Erin Evans, Dana M. Freund, Veronica M. Sondervan, Jerry D. Cohen, Adrian D. Hegeman^*^

*** Correspondence:** Adrian D. Hegeman: hegem007@umn.edu

## Supplementary Tables

**Table S3**. A compilation of pool size and turnover adjusted pool sizes for both the active and inactive pools for each experiment. Pool sizes are given in μmol/mg sample extract residue and turnover adjusted pool sizes are in units of μmol/mg sample extract residue/hour

| **Amino Acid** | **Experiment** | **Pool Size** | **Pool Size (Active)** | **Turnover Adjusted Pool Size** | **Turnover Adjusted Pool Size (Active)** |
| --- | --- | --- | --- | --- | --- |
| **Alanine** | Light With Sucrose | 0.0054 | 0.0026 | 0.0046 | 0.0040 |
|  | Light Without Sucrose | 0.0031 | 0.0027 | 0.0015 | 0.0012 |
|  | Dark With Sucrose | 0.0375 | 0.0318 | 0.0011 | 0.0010 |
| **Aspartic Acid** | Light With Sucrose | 0.0142 | 0.0108 | 0.0164 | 0.0125 |
|  | Light Without Sucrose | 0.0057 | 0.0051 | 0.0025 | 0.0022 |
|  | Dark With Sucrose | 0.0117 | 0.0082 | 0.0029 | 0.0020 |
| **Glutamate** | Light With Sucrose | 0.0279 | 0.0248 | 0.0196 | 0.0174 |
|  | Light Without Sucrose | 0.0100 | 0.0087 | 0.0040 | 0.0034 |
|  | Dark With Sucrose | 0.0166 | 0.0117 | 0.0054 | 0.0038 |
| **Phenylalanine** | Light With Sucrose | 0.0015 | 0.0010 | 0.0006 | 0.0004 |
|  | Light Without Sucrose | 0.0006 | 0.0005 | 0.0001 | 0.0001 |
|  | Dark With Sucrose | 0.0028 | 0.0011 | 0.0003 | 0.0001 |
| **Glycine** | Light With Sucrose | 0.0004 | 0.0003 | 0.0003 | 0.0002 |
|  | Light Without Sucrose | 0.0003 | 0.0002 | 0.0001 | 0.0001 |
|  | Dark With Sucrose | ------- | ------- | ------- | ------- |
| **Isoleucine/ Leucine** | Light With Sucrose | 0.0006 | 0.0004 | 0.0003 | 0.0002 |
|  | Light Without Sucrose | 0.0002 | 0.0001 | 3.0E-05 | 1.00E-05 |
|  | Dark With Sucrose | 0.0014 | 0.0003 | 0.0003 | 0.0001 |
| **Lysine** | Light With Sucrose | 0.0008 | 0.0007 | 0.0001 | 0.0001 |
|  | Light Without Sucrose | 0.0002 | 0.0002 | 3.0E-05 | 3.00E-05 |
|  | Dark With Sucrose | 0.0101 | 0.0020 | 0.0004 | 0.0001 |
| **Asparagine** | Light With Sucrose | 0.2282 | 0.1503 | 0.0233 | 0.0153 |
|  | Light Without Sucrose | 0.0844 | 0.0609 | 0.0216 | 0.0156 |
|  | Dark With Sucrose | 0.0400 | 0.0371 | 0.0017 | 0.0016 |
| **Proline** | Light With Sucrose | 0.0010 | 0.0007 | 0.0004 | 0.0003 |
|  | Light Without Sucrose | 0.0005 | 0.0002 | 0.0001 | 0.0001 |
|  | Dark With Sucrose | 0.0022 | 0.0005 | 0.0002 | 5.0E-05 |
| **Glutamine** | Light With Sucrose | 0.0704 | 0.0679 | 0.0823 | 0.0794 |
|  | Light Without Sucrose | 0.0310 | 0.0299 | 0.0253 | 0.0244 |
|  | Dark With Sucrose | 0.0512 | 0.0384 | 0.0770 | 0.0578 |
| **Arginine** | Light With Sucrose | 0.0007 | 0.0006 | 0.0002 | 0.0002 |
|  | Light Without Sucrose | 0.0004 | 0.0003 | 0.0001 | 0.0001 |
|  | Dark With Sucrose | 0.0488 | 0.0293 | 0.0011 | 0.0006 |
| **Serine** | Light With Sucrose | 0.0071 | 0.0058 | 0.0060 | 0.0049 |
|  | Light Without Sucrose | 0.0056 | 0.0047 | 0.0020 | 0.0017 |
|  | Dark With Sucrose | 0.0159 | 0.0087 | 0.0005 | 0.0003 |
| **Threonine** | Light With Sucrose | 0.0021 | 0.0015 | 0.0005 | 0.0004 |
|  | Light Without Sucrose | 0.0010 | 0.0007 | 0.0001 | 0.0001 |
|  | Dark With Sucrose | 0.0032 | 0.0014 | 0.0001 | 5.0E-05 |
| **Tryptophan** | Light With Sucrose | ------- | ------- | ------- | ------- |
|  | Light Without Sucrose | 0.0001 | 0.0001 | 8.06E-06 | 5.60E-06 |
|  | Dark With Sucrose | 0.0014 | 0.0008 | 5.0E-05 | 3.0E-05 |

**
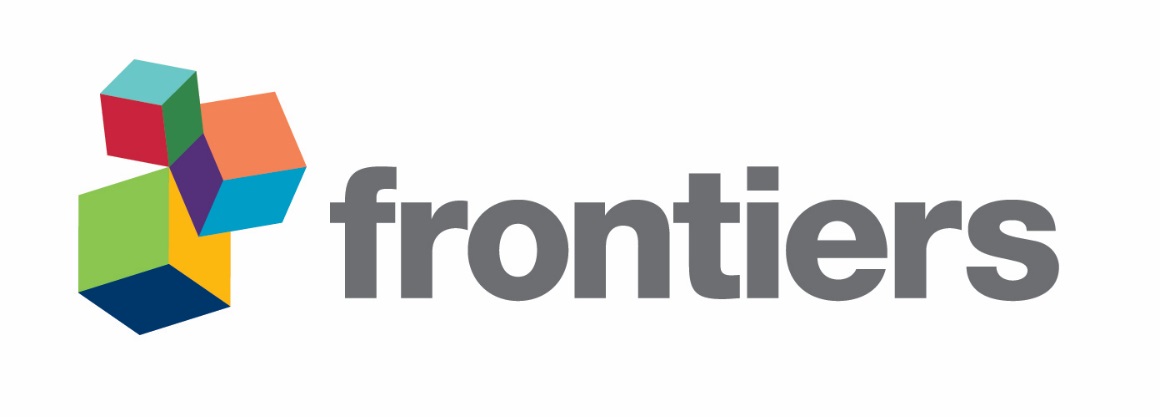
**
