# Supplementary material for: Metabolic Patterns in Spirodela polyrhiza Revealed by 15N Stable Isotope Labeling of Amino Acids in Photoautotrophic, Heterotrophic, and Mixotrophic Growth Conditions
Source: Front Chem. 2018 May 31;6:191. doi: 10.3389/fchem.2018.00191 (PMC5990592; doi:10.3389/fchem.2018.00191)
Supplement: Supplementary file 4 [file Table_4.DOCX]

Supplementary Material

Metabolic patterns in *Spirodela polyrhiza* revealed by ^15^N stable isotope labeling of amino acids in photoautotrophic, heterotrophic, and mixotrophic growth conditions

Erin Evans, Dana M. Freund, Veronica M. Sondervan, Jerry D. Cohen, Adrian D. Hegeman^*^

*** Correspondence:** Adrian D. Hegeman: hegem007@umn.edu

## Supplementary Tables

**Table S4**. P-values from pairwise significance tests between each experiment

| **Pool Size** | **A** | **D** | **E** | **F** | **G** | **I/L** | **K** |
| --- | --- | --- | --- | --- | --- | --- | --- |
| Light With Sucrose/Light Without Sucrose |  | 0.022 | 0.022 | 0.043 |  | 0.021 |  |
| Light With Sucrose/Dark With Sucrose | **0.014** |  |  |  | ---- |  | 0.034 |
| Light Without Sucrose/Dark With Sucrose | **0.010** |  |  | 0.020 | ---- | 0.041 | 0.025 |
|  | **N** | **P** | **Q** | **R** | **S** | **T** | **W** |
| Light With Sucrose/Light Without Sucrose | **0.019** | 0.033 | 0.036 |  |  |  | ---- |
| Light With Sucrose/Dark With Sucrose |  |  |  | 0.024 |  |  | ---- |
| Light Without Sucrose/Dark With Sucrose | 0.038 |  |  | 0.023 |  |  | **0.016** |
|  |  |  |  |  |  |  |  |
| **Pool Size (Active)** | **A** | **D** | **E** | **F** | **G** | **I/L** | **K** |
| Light With Sucrose/Light Without Sucrose |  | 0.044 | 0.020 |  |  |  |  |
| Light With Sucrose/Dark With Sucrose | **0.012** |  |  |  | ---- |  |  |
| Light Without Sucrose/Dark With Sucrose | 0.022 |  |  |  | ---- |  | 0.030 |
|  | **N** | **P** | **Q** | **R** | **S** | **T** | **W** |
| Light With Sucrose/Light Without Sucrose | 0.025 | **0.012** | 0.036 |  |  |  | ---- |
| Light With Sucrose/Dark With Sucrose |  |  |  | 0.024 |  |  | ---- |
| Light Without Sucrose/Dark With Sucrose |  |  |  | 0.023 |  |  | **0.017** |
|  |  |  |  |  |  |  |  |
| **Turnover Corrected Pool Size** | **A** | **D** | **E** | **F** | **G** | **I/L** | **K** |
| Light With Sucrose/Light Without Sucrose |  |  |  |  |  |  |  |
| Light With Sucrose/Dark With Sucrose | 0.047 |  |  |  | ---- |  | 0.040 |
| Light Without Sucrose/Dark With Sucrose |  |  |  | **0.019** | ---- | 0.039 | 0.030 |
|  | **N** | **P** | **Q** | **R** | **S** | **T** | **W** |
| Light With Sucrose/Light Without Sucrose |  | 0.006 |  |  | **0.012** |  | ---- |
| Light With Sucrose/Dark With Sucrose |  | 0.047 |  |  |  |  | ---- |
| Light Without Sucrose/Dark With Sucrose | **0.006** |  | 0.048 | 0.033 | 0.021 |  | **0.019** |
|  |  |  |  |  |  |  |  |
| **Turnover Corrected Pool Size (Active)** | **A** | **D** | **E** | **F** | **G** | **I/L** | **K** |
| Light With Sucrose/Light Without Sucrose |  |  |  |  | 0.049 |  |  |
| Light With Sucrose/Dark With Sucrose |  |  |  | 0.025 | ---- |  |  |
| Light Without Sucrose/Dark With Sucrose |  |  |  | 0.048 | ---- |  |  |
|  | **N** | **P** | **Q** | **R** | **S** | **T** | **W** |
| Light With Sucrose/Light Without Sucrose |  |  |  |  | **0.014** |  | ---- |
| Light With Sucrose/Dark With Sucrose |  |  |  |  |  |  | ---- |
| Light Without Sucrose/Dark With Sucrose | **0.001** |  |  | 0.036 | **0.015** |  | 0.023 |

**
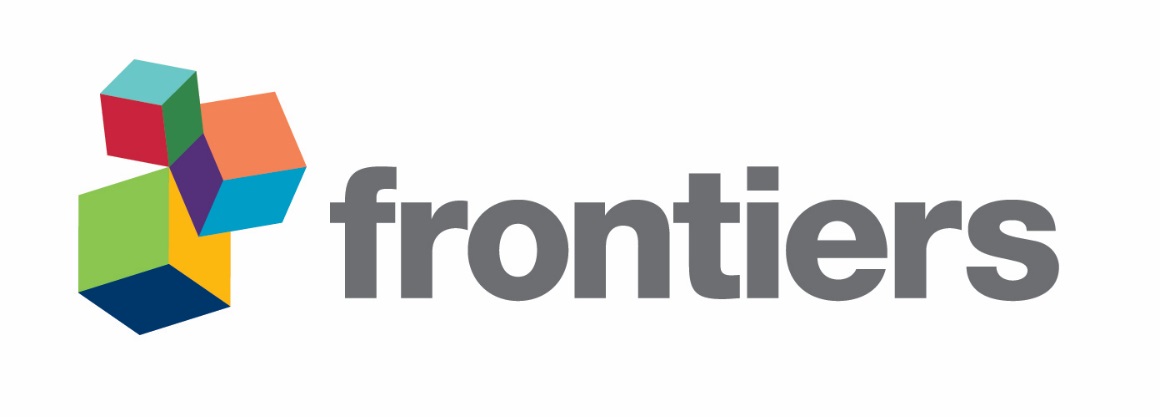
**
